# Multidimensional iron dysmetabolism and gut-brain axis interactions in restless legs syndrome: from peripheral deficiency to central dysregulation

**DOI:** 10.3389/fnagi.2026.1942435

**Published:** 2026-09-03

**Authors:** Ziyang Liu, Yunsong Zhang, Yating Li, Ting Li, Jianbo Jia, Tao Cai

**Affiliations:** 1 School of Sports Medicine and Health, Chengdu Sport University, Chengdu, China; 2 School of Pharmacy, Southwest Medical University, Luzhou, China; 3 Gulin County Hospital of Traditional Chinese Medicine, Luzhou, China; 4 Sports Medicine Key Laboratory of Sichuan Province, Chengdu, China; 5 Affiliated Sports Hospital of Chengdu Sport University, Chengdu, China; 6 Institute of Sports Medicine and Health, Chengdu Sport University, Chengdu, China

**Keywords:** blood-brain barrier, gut microbiota, individualized treatment, iron metabolic impairment, neurotransmitters, restless legs syndrome

## Abstract

Restless Legs Syndrome (RLS) is a sensorimotor neurological disorder characterized by an irresistible urge to move the lower limbs. Although traditional paradigms attribute its pathogenesis to peripheral iron deficiency, many clinically diagnosed patients present with normal peripheral iron indicators yet exhibit central iron homeostatic dysregulation. This discrepancy highlights a distinct decoupling between peripheral iron stores and central iron availability. The RLS-associated iron metabolic impairment is inherently a complex pathological network orchestrated by multidimensional processes, including intestinal absorption, systemic distribution, blood-brain barrier (BBB) transport, and intracellular utilization. Crucially, gut dysbiosis-induced low-grade enterogenous inflammation may activate the IL-6/hepcidin pathway, thereby sequestering systemically available iron; this mechanism represents a pivotal upstream driver of central iron insufficiency. Consequently, impaired cerebral iron utilization disrupts the physiological equilibrium of neurotransmitter systems—namely dopamine, adenosine, and glutamate—ultimately triggering the sensorimotor symptoms and sleep disturbances characteristic of RLS. Therefore, clinical management should shift away from relying solely on isolated peripheral indicators toward implementing individualized iron supplementation phenotyped by specific absorption, distribution, or transport mechanisms. Looking forward, microecological interventions utilizing prebiotics or short-chain fatty acids (SCFAs) to mitigate systemic inflammation and ameliorate the intestinal environment hold substantial promise as a synergistic therapeutic strategy for RLS.

## 1 Introduction

Restless Legs Syndrome (RLS) is a sensorimotor neurological disorder characterized by an irresistible urge to move the lower limbs, with iron metabolism dysregulation serving as one of the most definitive clues in its pathogenesis (Allen et al., 2003; Earley et al., 2014). Conventionally, RLS has been directly linked to systemic iron deficiency; indeed, patients exhibiting peripheral iron depletion often show significant symptomatic relief following iron supplementation (Trotti and Becker, 2019). However, peripheral iron status alone cannot fully account for RLS manifestation. In clinical practice, many patients present with serum iron and ferritin levels within the normal range, yet cerebrospinal fluid (CSF) analysis, neuroimaging, and neuropathological studies consistently reveal significant disruptions in central nervous system (CNS) iron homeostasis (Allen, 2015; Connor et al., 2003; Earley et al., 2000).

This disconnect between peripheral iron stores and actual central iron availability suggests that RLS-associated iron metabolic dysfunction is not merely a matter of total body depletion, but rather a complex pathological network involving iron absorption, mobilization, cross-barrier transport, and intracellular utilization (Gossard et al., 2021; Nemeth and Ganz, 2021). From dietary iron uptake by duodenal epithelium and systemic distribution mediated by the hepcidin-ferroportin axis, to the controlled endothelial transport of iron across the blood-brain barrier (BBB), impairment at any of these nodes can lead to a localized scarcity of bioavailable iron within the brain parenchyma—such as in the substantia nigra—thereby disrupting the physiological equilibrium of dopamine, adenosine, and glutamate neurotransmitter systems (Clemens et al., 2025; Cortés et al., 2019). Simultaneously, the gut microbiota, serving as a critical nexus for regulating host metabolism and immunity, not only competes for intestinal iron resources and reshapes the absorptive environment but may also alter systemic iron partitioning by inducing low-grade gut-derived inflammation, thereby emerging as a key upstream factor influencing cerebral iron supply (Choi and Bessman, 2025; Wang et al., 2026).

In the current era of rapidly advancing systems biology, resolving this pathological heterogeneity across the gut-brain axis increasingly requires the support of frontier technologies (Cryan et al., 2019; Valles-Colomer et al., 2019). To precisely delineate the dynamic distribution of iron across various cell types, future pathological investigations will heavily rely on multimodal data integration strategies. Examples include utilizing single-cell RNA sequencing to uncover impaired iron transport signaling between BBB endothelial cells and glial cells, employing spatial transcriptomics to map the aberrant expression profiles of iron-metabolism genes within the microenvironment of specific brain regions, and leveraging algorithmic molecular modeling to predict the modulatory effects of microbial metabolites on intracellular iron pools (Yang et al., 2022). Based on this multidimensional mechanistic perspective, this article will systematically review the pathological link between peripheral iron distribution and central iron utilization deficits in RLS, delve into the potential role of the gut microbiota in regulating iron metabolic pathways, and propose more precise clinical assessment and targeted therapeutic strategies tailored to the heterogeneous characteristics of iron metabolism in RLS.

## 2 Iron metabolism dysregulation in RLS

Iron metabolism dysregulation stands as one of the most definitive pathophysiological hallmarks in the investigation of RLS (Palsa et al., 2026). A subset of patients presents with diminished serum ferritin, decreased transferrin saturation, or other manifestations of systemic iron deficiency, and their symptoms frequently alleviate following iron supplementation (Earley et al., 2024; Short et al., 2024). However, systemic iron status alone fails to fully account for the pathogenesis of RLS. Clinically, a substantial proportion of patients exhibit normal serum iron and ferritin levels, yet CSF analyses, neuroimaging, and neuropathological studies consistently reveal central iron homeostatic abnormalities (Connor et al., 2011; Earley et al., 2000; Li et al., 2016). Taken together, RLS-associated iron metabolic dysfunction is not merely a reflection of absolute systemic iron depletion. Instead, it is highly likely to involve a multi-node pathological network spanning intestinal absorption, circulatory entry, BBB penetration, and ultimately, cellular uptake and utilization within the brain (Palsa et al., 2026).

The brain capillary endothelial cells precisely regulate cerebral iron influx via the transferrin receptor (TfR) system, with apotransferrin (Apo-Tf) and holotransferrin (Holo-Tf) signaling pathways dynamically modulating the transport process. The translocation of iron across the BBB necessitates a two-step transport across the luminal and abluminal membranes; consequently, an impairment at either node can culminate in insufficient cerebral iron supply. Any bottleneck along these pathways can generate a discrepancy between peripheral iron biomarkers and central iron status (Manolopoulos et al., 2024). Given these characteristics, the International Restless Legs Syndrome Study Group (IRLSSG) recommends a comprehensive evaluation of serum iron, ferritin, total iron-binding capacity (TIBC), and TSAT in RLS patients, guiding the choice between oral or intravenous iron supplementation based on their specific iron metabolic profiles (Kim et al., 2023). Current clinical practice also suggests that relying solely on serum ferritin to assess iron status in RLS is insufficient; the bioavailability of iron and its efficiency of transport into the CNS appear to carry equal clinical significance (Kim et al., 2023). Furthermore, the elevated ferritin along with downregulated transferrin receptors observed in neuron-derived extracellular vesicles (NDEVs) implies an impairment in neuronal iron uptake, underscoring that peripheral indices fail to mirror the true state of central iron utilization (Manolopoulos et al., 2024).

### 2.1 Systemic iron homeostasis: intestinal absorption and circulatory mobilization

Systemic iron status is fundamentally dictated by the absorption of dietary iron and the mobilization of stored iron into the circulation. Non-heme dietary iron is predominantly absorbed in the proximal duodenum, where it is reduced and transported into enterocytes via divalent metal transporter 1 (DMT1). It is subsequently exported into the bloodstream by ferroportin (FPN) and bound to transferrin for systemic distribution (Okazaki, 2024; Woloshun et al., 2022). Consequently, the pool of bioavailable circulating iron depends not only on dietary intake and mucosal absorption, but also on the efficiency of iron release from enterocytes and macrophages into the bloodstream (Fisher et al., 2025; Qiu et al., 2025).

Systemic iron availability is further sustained by the efficient recycling of iron from senescent erythrocytes and the mobilization of hepatic iron stores. Reticuloendothelial macrophages phagocytose aged red blood cells, degrade hemoglobin, and export the liberated iron back into the circulation via FPN (Ganz, 2011). Hepatocytes, which store iron in the form of ferritin and hemosiderin, similarly rely on FPN-mediated efflux to release iron when systemic demand increases (Nemeth and Ganz, 2021). The hepatic peptide hormone hepcidin serves as the master negative regulator of both processes: upon binding to FPN, hepcidin triggers its internalization and lysosomal degradation, thereby blocking iron export from macrophages, hepatocytes, and duodenal enterocytes alike. Consequently, hepcidin upregulation not only limits dietary iron absorption but also suppresses the mobilization of recycled and stored iron, leading to a state of functional iron deficiency wherein tissue iron reserves remain intact but circulating bioavailable iron is restricted (Ganz, 2011; Nemeth and Ganz, 2021). Hepcidin serves as the master regulator of this process; its upregulation triggers the endocytosis and degradation of ferroportin, thereby restricting both intestinal iron absorption and the release of stored iron into the circulation (Katsarou et al., 2021). Thus, systemic iron insufficiency can stem either from a genuine depletion of body iron stores or from restricted mobilization despite preserved iron reserves (Tawfik et al., 2024).

Despite the well-characterized roles of intestinal absorption and reticuloendothelial iron recycling in systemic iron homeostasis, direct evidence for primary defects in these processes specifically in RLS patients remains limited. Most studies to date have focused not on absorption or recycling per se, but on the hepcidin-ferroportin axis as a potential mediator of impaired iron mobilization. In drug-free patients with primary RLS and normal ferritin levels (>50 ng/mL), serum hepcidin levels were found to be significantly higher than in controls and were positively correlated with periodic limb movements during sleep (Dauvilliers et al., 2018). Importantly, this hepcidin elevation occurred in the absence of group differences in ferritin levels, suggesting that the abnormality lies not in iron storage depletion but in restricted iron release from macrophages and hepatic stores into the circulation. The IRLSSG task force guidelines similarly emphasize that peripheral iron assessment in RLS should extend beyond ferritin to include transferrin saturation and clinical context, as ferritin alone may be confounded by inflammation and does not capture the dynamic status of iron mobilization (Allen et al., 2018). Thus, while direct evidence for impaired intestinal absorption in RLS is scarce, the available data support a model in which hepcidin-mediated functional iron deficiency—characterized by adequate stores but restricted mobilization—contributes to the peripheral-central iron mismatch in a subset of patients (Allen et al., 2018; Dauvilliers et al., 2018).

Peripheral iron abnormalities in RLS patients can be broadly categorized into two phenotypes. The first group presents with absolute iron deficiency—commonly driven by inadequate dietary intake, chronic blood loss, pregnancy, or gastrointestinal malabsorption—typically characterized by depleted iron stores and reductions in serum ferritin or TSAT (Short et al., 2024; Winkelman and Wipper, 2026). The second group, however, does not necessarily exhibit a profound reduction in total iron reserves. Inflammatory responses and elevated hepcidin levels can suppress ferroportin-mediated iron export, causing iron to be sequestered within enterocytes and macrophages, which selectively diminishes the circulating iron pool available for tissue utilization (Nemeth and Ganz, 2021). RLS-related studies have observed elevated hepcidin levels in patients without a concurrent decline in ferritin (Dauvilliers et al., 2018)—a pattern highly consistent with restricted iron mobilization and allocation rather than pure reservoir exhaustion.

Consequently, serum ferritin alone cannot fully characterize the iron metabolic status of RLS patients. While ferritin primarily reflects body iron stores, it also functions as an acute-phase reactant susceptible to confounding by inflammation, obesity, and chronic comorbidities (Brzezinski et al., 2024; Urbanski et al., 2024). In a state of low-grade inflammation, ferritin levels may remain normal or even elevated, yet hepcidin-mediated restriction of iron release can still induce a decline in TSAT and reduce circulating bioavailable iron (Cacoub et al., 2022). Assessment of peripheral iron status in patients with RLS should extend beyond the evaluation of storage iron to include the capacity for iron mobilization into the systemic circulation. A comprehensive diagnosis requires the synthesis of serum ferritin, transferrin saturation, and pertinent clinical background (Allen et al., 2018).

### 2.2 CNS iron homeostasis: multilevel spatial heterogeneity and cellular utilization deficits

Iron distribution within the brain is remarkably heterogeneous, with distinct anatomical regions and cell types exhibiting discrete iron demands and regulatory mechanisms. The CNS is segregated from the systemic circulation by the blood-brain barrier and the blood-CSF barrier, making the replenishment and turnover of cerebral iron tightly restricted. Peripheral iron levels, therefore, do not directly reflect central iron status; even in the presence of normal circulatory indices, specific brain regions or cells may experience functional iron deficiency due to constraints in iron uptake, transport, or utilization (Earley et al., 2000).

In RLS, neuroimaging studies using quantitative susceptibility mapping (QSM) have revealed that brain iron deficiency is not a diffuse, global phenomenon, but rather exhibits pronounced regional specificity. A 7-tesla QSM study found that RLS patients showed significantly decreased magnetic susceptibility in the thalamus and dentate nucleus compared to healthy controls, and magnetic susceptibility in the substantia nigra (SN) was significantly correlated with periodic limb movements during sleep (PLMS) (Li et al., 2016). Notably, while no significant difference was observed in the SN at the group level, magnetic susceptibility in the SN was significantly correlated with PLMS. This correlation suggests that regional iron deficiency in the SN may be particularly relevant to the motor manifestations of RLS, even when global reductions are not apparent. These imaging findings underscore that the cerebral pathology of RLS is not a uniform depletion of brain iron, but rather a spatially heterogeneous dysregulation. Emerging QSM techniques hold promise for distinguishing between ferritin-bound storage iron and other paramagnetic iron species, which may provide more nuanced insights into the subcellular iron allocation deficits hypothesized in RLS.

At the cellular level, iron uptake, storage, and efflux within brain tissue rely on the coordinated actions of multiple proteins, including transferrin receptors, DMT1, ferritin, and ferroportin (Connor et al., 2011). However, bulk tissue-level iron signals—whether from MRI or postmortem tissue analysis—cannot serve as a proxy for evaluating the metabolic status of iron processing within individual neurons. Recent advances in NDEVs have provided unprecedented insight into neuronal iron metabolism in living RLS patients. In a case-control study of 71 women with iron deficiency anemia, Manolopoulos et al. (2024) found that RLS patients exhibited elevated ferritin and decreased TfR levels in NDEVs compared to non-RLS controls. This pattern suggests a diminished neuronal capacity for iron uptake and a potential mechanism whereby neurons deplete their intracellular bioavailable iron by secreting ferritin-loaded vesicles. Critically, these NDEVs abnormalities were observed in RLS patients despite the presence of systemic iron deficiency, indicating that neuronal iron dysregulation can be dissociated from peripheral iron status (Manolopoulos et al., 2024).

Furthermore, the functional consequences of iron deficiency depend on its sub-cellular allocation. Iron is indispensable for mitochondrial oxidative metabolism, driving iron-sulfur cluster and heme synthesis, and is inextricably linked to respiratory chain integrity and ATP generation. Based on the central role of iron in bioenergetics, it has been hypothesized that when intracellular iron trafficking is disrupted, mitochondria may experience an organelle-specific “relative iron deficiency” despite adequate total tissue iron, subsequently undermining oxidative phosphorylation and redox homeostasis (Valle-León et al., 2026). Supporting this hypothesis, postmortem studies of RLS substantia nigra have revealed increased mitochondrial ferritin in neuromelanin-containing neurons, along with increased numbers of mitochondria, whereas cytosolic H-ferritin was decreased (Snyder et al., 2009). These findings are consistent with the hypothesis that energy insufficiency in these neurons may be involved in the pathogenesis of RLS (Snyder et al., 2009). However, direct *in vivo* evidence for this specific cascade in RLS remains limited, and this mechanism should currently be viewed as a compelling theoretical model rather than an established pathological fact (Valle-León et al., 2026). A more grounded consensus is that central iron abnormalities in RLS are multifaceted—encompassing barrier transport, cellular uptake, and intracellular utilization—and cannot be simplified as a mere reduction in localized iron content (Manolopoulos et al., 2024).

### 2.3 The blood-brain barrier as a gatekeeper for central iron supply

Systemic circulating iron cannot freely enter the brain parenchyma. The BBB, formed by brain microvascular endothelial cells (BMECs) with tight junctions and specialized transport machinery, effectively segregates central iron metabolism from the periphery, rendering the cerebral iron supply critically dependent on regulated transendothelial transport rather than passive fluctuations in serum iron levels (Khan et al., 2018; Nielsen et al., 2023). Circulating iron, predominantly bound to transferrin (Tf), is captured by TfR1 on the luminal surface of BMECs. Following endosomal acidification, iron is liberated from Tf into the cytosolic pool; a portion is sequestered within endothelial ferritin, while the remainder is exported across the abluminal membrane into the brain interstitium for uptake by neurons and glia (Baringer et al., 2023; Guo et al., 2026). FPN is implicated in this abluminal efflux, while hephaestin, ceruloplasmin, and localized hepcidin signaling may jointly modulate iron transfer from the endothelium into the brain parenchyma (Baringer et al., 2023; Guo et al., 2026). Importantly, this process is not a passive conduit but an actively regulated system, as the expression of TfR1 on BMECs is modulated by intracellular iron status through iron-regulatory proteins (IRPs), allowing the BBB to respond to both systemic and local iron demands (You et al., 2022).

**FIGURE 1:**
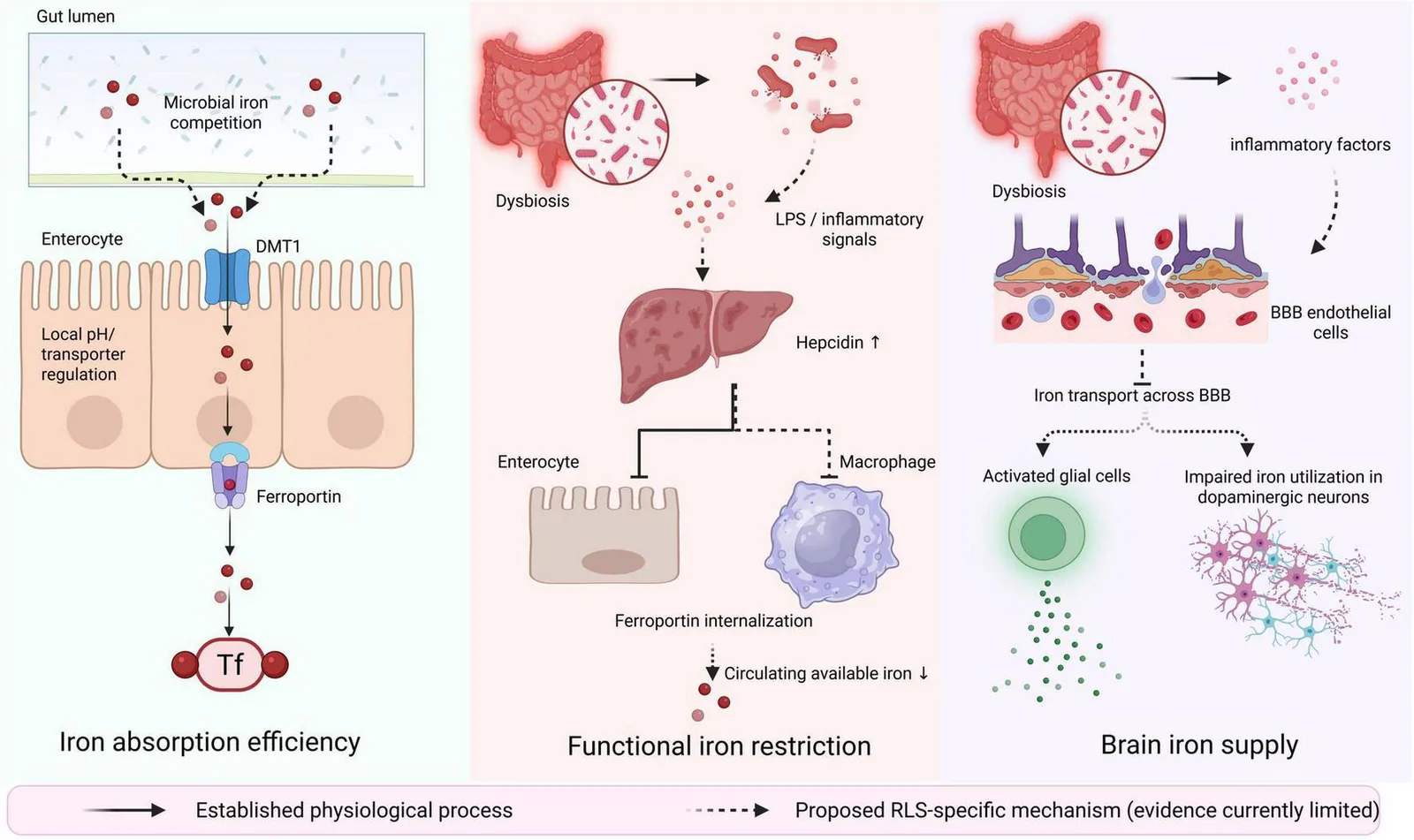
Schematic illustration of the multi-node pathological network underlying the peripheral-central “iron mismatch” in Restless Legs Syndrome (RLS). **(Left)** Peripheral iron status is determined by dietary iron intake, gut microbiota competition, and absorption via enterocyte divalent metal transporter 1 (DMT1) and ferroportin. Reduced circulating bioavailable iron can arise from absolute iron deficiency or functional iron restriction. **(Middle)** The blood-brain barrier (BBB) transport disconnection illustrates the central paradox in RLS: peripheral iron levels are normal, yet the brain fails to acquire adequate iron. Proposed RLS-specific mechanisms include cytokine-induced hepcidin upregulation, ferroportin internalization, and disrupted TfR1 trafficking in BBB endothelial cells, leading to impaired endosomal iron release and reduced iron uptake by the brain parenchyma. **(Right)** Brain iron homeostasis at the dopaminergic neuronal level. Iron imported via TfR1 enters the labile iron pool to support mitochondrial ATP production and dopamine synthesis. RLS-specific hypotheses highlight that impaired intracellular iron utilization and regional iron deficiency (particularly in the substantia nigra) can disrupt neurotransmitter systems.

In RLS, multiple lines of evidence point to intrinsic dysfunction of BBB iron transport. CSF from untreated RLS patients has been shown to significantly reduce iron uptake in cultured human BMECs and interfere with the regulation of TfR1 expression (Palsa et al., 2026). This finding provides a direct, *in vitro*-model-based pathophysiological explanation for the clinical paradox wherein central iron supply is restricted despite normal peripheral iron levels. Furthermore, elevated ferritin and decreased transferrin receptor levels in NDEVs from RLS patients suggest impaired neuronal iron uptake and intracellular utilization—a phenomenon that can exist independently of peripheral iron deficiency (Manolopoulos et al., 2024). These observations collectively indicate that the BBB in RLS is not merely a passive bystander but may actively contribute to the central iron insufficiency through defective receptor-mediated transport, impaired abluminal efflux, or both. It is important to note, however, that while inflammatory mediators and oxidative stress are known to impair BMEC barrier function and tight junction integrity, whether these changes definitively reduce TfR1-mediated iron uptake in RLS requires specific validation (VanHook, 2024; Xingi et al., 2023). Currently, no direct evidence links gut microbiota alterations to impaired BBB iron transport specifically in RLS (Lehmann et al., 2023). Although gut-derived inflammation can activate systemic inflammatory pathways and elevate hepcidin levels, which in turn may influence systemic iron distribution (Davaanyam et al., 2023), a causal chain from intestinal dysbiosis to BBB iron transport dysfunction has not been established in RLS patients. Experimental studies have shown that astrocyte-derived hepcidin can regulate FPN1 on BMECs, affecting iron release into the brain parenchyma (Guo et al., 2026), but whether this mechanism is triggered by gut-derived signals in RLS remains speculative, At this stage, a more prudent conclusion is that gut microbiota alterations, BBB iron transport abnormalities, and impaired neuronal iron utilization may be interconnected at the level of systemic inflammation and iron regulation, but the precise causal sequence in RLS remains to be elucidated (Manolopoulos et al., 2024). The multi-node pathological network and transportation bottlenecks of iron dysmetabolism in RLS are schematically summarized in Figure 1.

Solid arrows indicate established physiological processes, whereas dashed arrows denote proposed RLS-specific mechanisms that currently have limited or circumstantial evidence.

Collectively, these pathways demonstrate that normal peripheral iron does not guarantee adequate brain iron, and that RLS iron dysmetabolism is a complex, multi-stage disorder rather than a simple total-body depletion.

## 3 Gut microbiota and the regulatory pathways of iron metabolism

The gut microbiota has emerged as a critical modulator of CNS function through the microbiota–gut–brain axis, a bidirectional communication system linking the gastrointestinal tract and the brain via neural, immune, endocrine, and metabolic pathways (Cryan et al., 2019; Khan et al., 2022). The gut microbiota communicates with the brain through multiple interconnected routes: immune signaling, wherein microbial components such as lipopolysaccharides (LPS) and microbial metabolites modulate systemic and central inflammatory responses (Macpherson et al., 2023); microbial metabolites, particularly SCFAs, tryptophan derivatives, and bile acids, which can cross the intestinal barrier, enter the circulation, and influence CNS functions including neuroinflammation, synaptic plasticity, and neurotransmitter metabolism (O’Riordan et al., 2022); intestinal barrier function, whose integrity is essential for preventing the translocation of luminal microbes and their products into the systemic circulation, thereby maintaining immune homeostasis and limiting neuroinflammation (Macpherson et al., 2023); and neural pathways, including the vagus nerve and the enteric nervous system, which provide direct anatomical routes for gut-derived signals to reach brainstem nuclei and higher brain centers (Cryan et al., 2019). These pathways are well-characterized in various neurological and neurodegenerative conditions, where gut dysbiosis has been implicated in disease pathogenesis through modulation of neuroinflammation, blood–brain barrier integrity, and glial function (Tan et al., 2022). Recent comprehensive reviews have further underscored the therapeutic potential of targeting the microbiota–gut–brain axis in neurodegenerative diseases, with particular emphasis on the roles of gut microbial metabolites in regulating microglial, astrocytic, and oligodendrocytic functions (Loh et al., 2024). In Huntington’s disease, emerging evidence has delineated three convergent mechanistic cascades through which dysbiosis may modify disease biology: short-chain fatty acid-driven HDAC and GPCR pathways, bile acid-dependent FXR/TGR5 circuits, and microbiota-regulated tryptophan metabolism encompassing serotonin/melatonin rhythms, indole-aryl hydrocarbon receptor immunomodulation, and kynurenine pathway neurotoxicity (Gu et al., 2026). Similarly, in amyotrophic lateral sclerosis, microbial metabolites have been shown to impact neurological function through the enteric nervous system, which serves as an intermediary interface linking luminal microbial signals to central nervous system vulnerability (Walton and Sun, 2026). However, direct evidence linking these specific microbiota–gut–brain axis mechanisms to the pathophysiology of RLS remains largely speculative and warrants systematic investigation, particularly regarding their potential roles in iron metabolism regulation and central iron availability (Manolopoulos et al., 2024).

Although there is currently no definitive evidence that gut microbiota alterations are a direct cause of iron metabolism abnormalities in RLS, the gut remains a critical upstream regulatory site. This rationale is based on three pillars: iron absorption occurs primarily in the gut, where luminal iron is utilized by both the host and microbiota; intestinal barrier integrity and inflammatory states modulate systemic iron distribution; and associations between gastrointestinal disorders and gut microbial dysbiosis have been consistently observed in RLS patients (Banihashemian et al., 2025; Khan et al., 2022; Montini et al., 2026). Given that these lines of evidence represent different levels of biological hierarchy, they cannot yet be synthesized into a validated causal chain. Therefore, this section explores whether the gut microbiota may contribute to the pathogenesis of RLS by influencing intestinal iron absorption, systemic distribution, and CNS iron availability.

### 3.1 Gut microbiota and luminal iron bioavailability

The intestine serves as both the primary site for host iron absorption and a critical niche for microbial colonization. Not all dietary iron is absorbed by the host; a portion remains available for microbial utilization. Many bacteria secrete high-affinity siderophores to sequester luminal iron, while others exploit transferrin- or lactoferrin-bound iron, creating an inherent resource competition between the host and the microbiome (Mayneris-Perxachs et al., 2022). Small intestinal bacterial overgrowth or microbial dysbiosis may reduce iron bioavailability through competitive uptake, potentially exacerbating peripheral iron deficiency in RLS patients who are already on the threshold of iron depletion or suffering from iron-deficiency anemia (Auerbach et al., 2025; Li et al., 2025; Liu et al., 2023).

The influence of the gut microbiota on iron absorption extends beyond direct competition for luminal iron. Animal studies have demonstrated differential expression of iron transport proteins—such as DMT1, DCYTB, and ferroportin—between germ-free and colonized states, suggesting that microorganisms regulate host iron absorption machinery (Noordine et al., 2024). Furthermore, microbial metabolic activity alters the local chemical environment. The production of SCFAs, lactate, and other organic acids lowers luminal pH, influencing the solubility and reduction state of iron, thereby modulating its uptake by intestinal epithelial cells (Wang and Zhou, 2025; Yan et al., 2024). Consequently, the efficiency of dietary iron absorption may vary significantly depending on the underlying microbial composition (Choi and Bessman, 2025; Sun et al., 2024).

Direct evidence in RLS remains limited. While recent studies have identified distinct gut microbiome signatures in RLS patients compared to those with insomnia and healthy controls, these findings do not confirm that microbial shifts directly impair iron absorption (Banihashemian et al., 2025; Khan et al., 2022; Montini et al., 2026). Clinical observations regarding SIBO and gastrointestinal symptoms suggest that some patients may experience increased bacterial load or mucosal dysfunction (Leite et al., 2024). Thus, it is reasonable to hypothesize that the gut microbiome alters iron bioavailability and epithelial transport efficiency, thereby impacting peripheral iron supply; however, this mechanism requires direct verification in RLS cohorts (Liu et al., 2024; Xiao et al., 2023).

A bidirectional relationship also exists between iron and the gut microbiota. Unabsorbed oral iron passes into the distal colon, where it acts as an environmental factor that reshapes microbial ecology (Celis et al., 2023). Elevated luminal iron exposure has been shown to promote the proliferation of certain pathobionts, often accompanied by oxidative stress and mucosal inflammation (Gu et al., 2025). This suggests that while the microbiota influences iron absorption, iron supplementation itself may modulate the gut environment. For RLS, this reciprocal interaction may contribute to suboptimal responses to oral iron therapy, a hypothesis that warrants further investigation in clinical treatment response studies (Dentand et al., 2024; Lai et al., 2022).

### 3.2 Gut-derived inflammation and hepcidin-mediated iron restriction

Intestinal dysfunction affects iron metabolism beyond the absorption phase. Persistent inflammation can alter systemic iron partitioning, preventing its mobilization into circulation even when iron stores are sufficient. Upon intestinal barrier disruption, microbial-derived molecules such as LPS enter the circulation, activating inflammatory signaling pathways—including TLR4/NF-κB—and triggering IL-6 release (Ma et al., 2023; Zheng et al., 2024). Subsequently, IL-6 promotes hepatic hepcidin synthesis via the JAK/STAT3 pathway (Faradina et al., 2023; Zhao et al., 2022). Elevated hepcidin binds to ferroportin, inducing its internalization and degradation (Charlebois et al., 2022; Traeger et al., 2022), which inhibits both intestinal iron absorption and macrophage iron recycling. This state, known as functional iron deficiency (FID), results in reduced iron availability for tissue utilization, despite potentially normal total body iron stores.

Clinical data in RLS support this model of restricted iron distribution. Studies have observed higher hepcidin levels in RLS patients compared to controls, in the absence of a corresponding decrease in ferritin; notably, hepcidin levels correlate more strongly with symptom severity and periodic limb movements than ferritin levels do (Kim et al., 2023; Nemeth and Ganz, 2023). This indicates that iron metabolism abnormalities in some patients are not solely attributable to depletion, but rather to impaired iron mobilization and release: stores are present, but hepcidin-mediated inhibition of ferroportin limits circulating iron availability (Walters et al., 2024). While the link between elevated hepcidin and impaired iron transport in RLS is established, there is no definitive evidence directly tracing this inflammatory response to gut dysbiosis, and other sources—such as adipokines from obesity or endothelial injury—cannot be excluded (Kim et al., 2023; Manolopoulos et al., 2024).

The clinical association between RLS and gastrointestinal disorders provides further clues. Given that SIBO and intestinal barrier dysfunction can coexist with chronic low-grade inflammation (Bushyhead and Quigley, 2022), it is plausible that gut-derived inflammation limits iron release via the IL-6–hepcidin axis. However, lacking studies that simultaneously correlate specific microbial dysbiosis, elevated hepcidin, and impaired iron utilization in RLS, it would be premature to assume this pathway applies to all patients with normal peripheral iron (Bushyhead and Quigley, 2022; Cui et al., 2025). At the current stage, a more prudent explanation is that a subset of RLS patients—particularly those presenting with intestinal barrier impairment or low-grade inflammation—may exhibit hepcidin-mediated restriction of iron release (Dauvilliers et al., 2025). This restriction leads to a divergence between the iron storage status reflected by ferritin and the actual bioavailability of circulating iron. Furthermore, this phenomenon may serve as a critical link in understanding why some patients experience insufficient central iron supply despite maintaining normal ferritin levels (Winkelman and Wipper, 2026).

### 3.3 Neurochemical consequences of central iron insufficiency

Building upon the gut-derived inflammatory and hepcidin-mediated pathways discussed above, the ultimate neurological manifestations of RLS arise from central iron insufficiency and its impact on neurotransmitter systems. While the gut microbiota and its metabolic products modulate systemic iron availability and peripheral inflammatory status (Xiao et al., 2023), it is the subsequent impairment of cerebral iron utilization that directly perturbs the neurochemical balance governing sensorimotor and sleep regulation (Cryan et al., 2019). Dopamine represents the most extensively studied pathway. Iron is essential for tyrosine hydroxylase, the rate-limiting enzyme in dopamine synthesis; consequently, brain iron deficiency potentially alters dopamine synthesis, release, and receptor regulation (Chen et al., 2025). Existing literature suggests that RLS is not merely a dopamine deficiency, but likely involves a rhythmic imbalance and compensatory changes within the dopaminergic system (Li et al., 2024; Silvani et al., 2023). Particularly at night, circadian-driven fluctuations may amplify these abnormalities, impairing sensorimotor integration and manifesting as the hallmark RLS features of nocturnal symptom exacerbation and relief through movement.

Dopaminergic dysfunction alone fails to fully account for the profound sleep fragmentation and hyperarousal seen in RLS patients. Recently, the adenosine system has garnered significant attention. In animal models of brain iron deficiency, downregulation of adenosine A1 receptors is observed; these receptors normally exert an inhibitory effect on dopamine and glutamate release (Franco et al., 2026). When this inhibition wanes, neurotransmitter release in the striatum and related brain regions shifts toward an excitatory state. Rodrigues et al. (2022) proposed that adenosine hypofunction resulting from brain iron deficiency is a fundamental basis for RLS neurotransmitter abnormalities. In this context, the imbalance between dopaminergic and glutamatergic systems in RLS may not be independent, but rather a synergistic manifestation of disinhibited sensorimotor networks and a reset of sleep-wake thresholds, driven by defective adenosinergic signaling (Sahay et al., 2023; Wiprich et al., 2022).

Enhanced glutamatergic activity further explains the hyperarousal and sleep-maintenance difficulties in RLS. Imaging studies have identified elevated thalamic glutamate/glutamine signals in RLS patients, which correlate with increased nocturnal wakefulness (Garcia-Borreguero et al., 2024). Thus, brain iron deficiency likely keeps the sensorimotor and sleep-wake systems in a state of chronic hyper-responsiveness by modulating adenosine’s regulation of dopaminergic and glutamatergic pathways (Franco et al., 2026).

**FIGURE 2:**
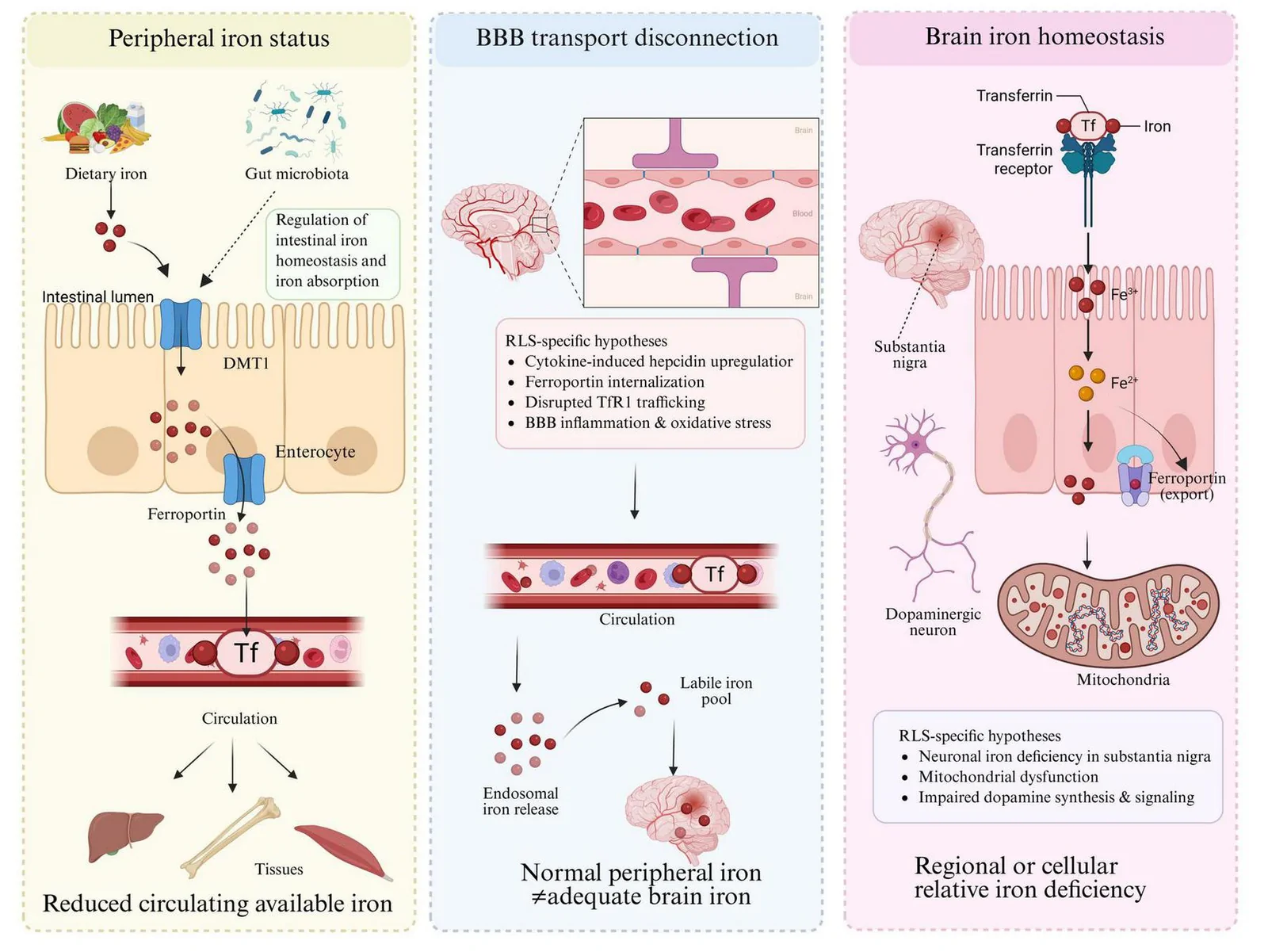
Schematic representation of the potential gut microbiota-mediated regulatory pathways of iron metabolism in Restless Legs Syndrome (RLS). **(Left)** Dietary iron enters the intestinal lumen as an independent source, followed by divalent metal transporter 1 (DMT1)-mediated uptake into enterocytes and ferroportin-dependent export into the circulation bound to transferrin. The gut microbiota is displayed as a separate modulatory entity, with dashed arrows indicating its regulatory influences on luminal iron bioavailability (via siderophore competition), enterocyte iron transport [via short-chain fatty acids (SCFAs) and other metabolites modulating DMT1 and ferroportin expression], and overall intestinal iron homeostasis. These regulatory influences are modulatory rather than prerequisite, and do not position dietary iron downstream of the gut microbiota. **(Middle)** Functional iron restriction: intestinal dysbiosis can trigger the release of LPS and other inflammatory signals into the systemic circulation. This activates the IL-6/hepcidin axis in the liver, causing hepcidin upregulation. Elevated hepcidin induces ferroportin internalization in enterocytes and macrophages, consequently reducing circulating available iron—a condition characteristic of functional iron deficiency despite adequate total body iron stores. **(Right)** Brain iron supply and neuroglial interplay: persistent inflammatory factors derived from intestinal dysbiosis may impair blood-brain barrier (BBB) endothelial cell integrity and function. While the exact molecular link remains speculative, this dysfunction may lead to impaired iron transport across the BBB, contributing to activated glial cell responses and ultimately disrupting iron utilization within dopaminergic neurons in the substantia nigra.

Collectively, the neurochemical disturbances described above represent the downstream consequence of the iron metabolic dysregulation cascade originating from gut–brain interactions. Therefore, the relationship between brain iron deficiency and RLS symptoms is not simply one of iron deficiency directly causing a specific symptom. A more plausible explanation is that impaired iron utilization disrupts the homeostatic balance among dopamine, adenosine, and glutamate, leading to elevated sensorimotor excitability while concurrently weakening the ability to maintain nocturnal sleep (Lai et al., 2022). The aforementioned factors—impaired intestinal iron absorption, inflammation-mediated iron restriction, and abnormal blood-brain barrier transport (Carson and Brittenham, 2023; Palsa et al., 2026), which can ultimately manifest as the characteristic motor urge, sensory abnormalities, and sleep disturbances of RLS only when they further compromise intracerebral iron utilization and disturb these neurotransmitter systems (Winkelman, 2022). Thus, the gut microbiota–iron metabolism axis, through its modulation of systemic iron bioavailability and inflammatory tone, may ultimately exert its influence on RLS symptomatology via these neurochemical pathways. The potential pathways are schematically illustrated in Figure 2.

This diagram illustrates how gut-derived signals may influence systemic iron availability and iron supply. Solid arrows represent established physiological processes; dashed arrows denote proposed RLS-specific mechanisms that currently rely on indirect or extrapolated evidence.

## 4 Mechanistic heterogeneity of iron metabolism in RLS and therapeutic strategies

The status of iron metabolism in patients with RLS exhibits profound heterogeneity (Elangovan et al., 2025). While a subset of patients presents with genuine depletion of iron stores primarily secondary to impaired dietary intake or intestinal absorption (Gaisinskaya et al., 2022), others exhibit preserved serum ferritin reserves but experience limited iron mobilization due to systemic inflammation and elevated hepcidin levels (Papastergiou et al., 2024). Furthermore, a distinct cohort demonstrates normal peripheral iron indices alongside central defects in iron transport or utilization (Ma et al., 2024). Consequently, identical clinical phenotypes may mask fundamentally divergent underlying disruptions in iron pathways (Kharel et al., 2024). Variations in response to iron supplementation must be contextualized within this framework: the correction of peripheral iron deficiency does not invariably equate to the synchronous restoration of CNS iron supply (Rao, 2024). Rather than superficially classifying iron therapy as simply effective or ineffective, clinical focus must shift toward identifying whether the metabolic bottleneck resides in absorption, systemic distribution, or central acquisition (Winter and Harris, 2023).

### 4.1 Pathophysiological phenotyping of iron dysregulation in RLS

Based on existing research, iron metabolism abnormalities in RLS patients may occur at different stages, though the dominant bottleneck varies among individuals. Some patients primarily exhibit insufficient iron supply, commonly due to reduced dietary intake, impaired gastrointestinal absorption, or low bioavailability of oral iron, often accompanied by decreased serum ferritin and transferrin saturation. For these patients, the core issue lies in the initial stage of systemic iron entry; therefore, improving iron intake, correcting malabsorption, and managing underlying gastrointestinal disorders theoretically offer a higher likelihood of restoring iron status (Winkelman and Wipper, 2026). The prevalence of RLS is 5–6 times higher in patients with iron deficiency anemia (IDA), and multiple open-label trials have confirmed that iron supplementation can alleviate symptoms (Short et al., 2024).

Other patients do not show significant depletion of iron stores; instead, their abnormalities primarily involve iron release and systemic distribution. Chronic inflammation or elevated hepcidin levels can inhibit ferroportin, causing iron to be sequestered within enterocytes and macrophages, thereby reducing circulating bioavailable iron. Studies have shown that oral iron supplementation may trigger a paradoxical rise in hepatic hepcidin, leading to ferroportin degradation. This causes absorbed iron to become trapped within enterocytes, hepatocytes, and macrophages, maintaining a state of systemic iron deficiency despite ongoing supplementation (Nemeth and Ganz, 2021). The evidence that elevated hepcidin levels in RLS patients correlate with symptom severity suggests that this mechanism of functional iron deficiency exists in a subset of patients. In such cases, simply increasing oral iron dosage may not sufficiently improve iron availability, as the rate-limiting factor is not insufficient intake, but the inability to effectively mobilize stored iron. If patients concurrently present with SIBO, IBS, or other chronic inflammatory states, the link between intestinal abnormalities and elevated hepcidin warrants further evaluation (Charlebois et al., 2022).

Still other patients exhibit largely normal peripheral iron indices, yet central iron abnormalities persist. Existing neuroimaging and pathological studies suggest that cerebral iron alterations in RLS do not manifest as uniform, global iron reduction, but more likely involve BBB transport defects, regional iron misdistribution, and impaired intracellular utilization (Manolopoulos et al., 2024; Palsa et al., 2026). The endothelial BBB is responsible for regulating cerebral iron uptake, and its dysfunction may directly contribute to RLS pathogenesis (Nielsen et al., 2023). Elevated ferritin levels and decreased TfR expression in NDEVs indicate that RLS patients suffer from impaired neuronal iron uptake and intracellular utilization—a phenomenon that can exist independently of peripheral iron deficiency (Manolopoulos et al., 2024). Iron dysregulation in specific brain regions, such as the substantia nigra, has also been confirmed to correlate with motor symptoms and reduced sleep efficiency (Wang et al., 2022). For these patients, whether increasing peripheral iron levels can improve symptoms depends on whether the iron can effectively cross the BBB and be utilized by neurons. Therefore, serum ferritin reflects only a portion of the complex landscape of RLS iron metabolism (Auerbach et al., 2025). Future efforts to identify the iron metabolic profiles of individual patients must integrate assessments of transferrin saturation, inflammation, hepcidin levels, gastrointestinal function, and brain iron-related imaging or biomarkers (Lee et al., 2025). Furthermore, this phenotyping framework may offer insights into augmentation, a paradoxical worsening of RLS symptoms associated with long-term dopaminergic therapy. Since augmentation is more frequent in patients with lower baseline iron stores and may involve D1 receptor sensitization, identifying the predominant iron metabolic bottleneck in an individual could inform strategies to mitigate this complication, such as optimizing iron status prior to or during dopaminergic treatment. To facilitate clinical application of this phenotyping framework, we summarize the key diagnostic features and matched therapeutic strategies in Table 1.

**TABLE 1:** Iron metabolism phenotypes in restless legs syndrome: diagnostic features and therapeutic implications.

Phenotype	Key laboratory findings	Predominant pathological bottleneck	Recommended intervention	Clinical considerations
Absolute iron deficiency	Serum ferritin < 50 ng/mL; TSAT < 20%; low serum iron	Inadequate intake, chronic blood loss, or malabsorption	Oral iron supplementation (ferrous salts); correct underlying cause	First-line if tolerated; response expected within weeks; monitor ferritin and TSAT
Functional iron deficiency	Ferritin normal or elevated (≥50 ng/mL); TSAT < 20%; hepcidin ↑; CRP ↑ (if inflammatory)	Hepcidin-mediated ferroportin internalization; iron trapped in stores	Intravenous iron (e.g., ferric carboxymaltose); consider anti-inflammatory measures if chronic inflammation present	Oral iron may be ineffective; IV iron bypasses hepcidin block; evaluate for SIBO/IBS or low-grade inflammation
Central transport/utilization deficit	Peripheral iron indices normal; CSF ferritin ↓ (or elevated NDEV ferritin with ↓ TfR); QSM may show regional brain iron reduction (thalamus, SN)	BBB receptor-mediated transport impairment; neuronal uptake or subcellular allocation defect	Intravenous iron (may benefit subset); no established specific therapy; research on BBB-targeted agents	Response to IV iron is variable; phenotyping requires CSF or NDEV biomarkers (investigational); consider neuroimaging (QSM) if available

### 4.2 Divergence in iron supplementation routes and therapeutic responses

The inconsistent response of RLS patients to iron therapy is primarily rooted in the route of administration. Oral iron requires gastrointestinal dissolution, enterocyte uptake, and ferroportin-mediated export, and its absorption efficiency is susceptible to factors such as gastric acidity, dietary composition, mucosal integrity, and hepcidin levels (Haase, 2022). For patients with gastrointestinal malabsorption or elevated hepcidin, even escalating oral iron doses may result in limited systemic delivery. Simultaneously, unabsorbed iron continues into the distal intestine. Previous studies on oral iron and the gut microbiota have found that high luminal iron exposure can alter microbial composition and increase the potential for local oxidative stress and mucosal inflammation (Xiao et al., 2023). Thus, for RLS patients requiring long-term oral iron therapy, poor treatment response is not always a matter of insufficient dosage; one must also consider absorptive conditions and gastrointestinal status.

Intravenous (IV) iron bypasses the gastrointestinal absorption pathway and can more rapidly increase circulating bioavailable iron, offering an advantage when oral iron is ineffective, poorly tolerated, or indicated by specific clinical criteria. Current RLS guidelines have integrated iron metabolism indices into clinical decision-making, recommending oral or IV iron based on serum ferritin, transferrin saturation, and clinical presentation. Among these, the efficacy of intravenous ferric carboxymaltose in moderate-to-severe RLS is supported by a substantial body of clinical research (Earley et al., 2024). Compared to oral iron, IV administration reduces the influence of intestinal absorption variability, but this does not guarantee the same degree of symptomatic relief for all patients.

The reason may lie in the fact that elevated peripheral iron levels are merely a prerequisite for restoring central iron supply. Circulating iron must still undergo transport across the BBB and complete cellular uptake, storage, and subcellular distribution within the brain (Guo et al., 2026). If the limiting step is primarily peripheral, symptoms are more easily improved following supplementation; however, if there are intrinsic abnormalities in BBB iron transport or central iron utilization, symptoms may not synchronize with the improvement of peripheral iron indices. Therefore, one cannot simplistically retro-infer a specific pathological site based solely on whether oral or IV iron is effective. Nevertheless, the efficacy differences between administration routes confirm that RLS iron metabolic disorders do not all occur at the same location. Future clinical studies should look beyond the normalization of serum ferritin and transferrin saturation and further focus on whether the correction of systemic iron metabolism can truly translate into sustained clinical benefit.

Furthermore, this multidimensional mechanistic framework offers a plausible explanation for the clinically recognized augmentation phenomenon associated with dopaminergic agents: central iron deficiency and homeostatic dysbalance render the dopamine, adenosine, and glutamate neurotransmitter systems susceptible to a fragile excitability threshold. Chronic exogenous dopaminergic stimulation may further exacerbate such rhythmic dysregulation and alter receptor sensitivity, paradoxically provoking more severe nocturnal motor urges and hyperarousal. This implies that, for patients with refractory RLS or those presenting with augmentation, reassessing iron metabolic status and mitigating non-specific central hyperexcitability could serve as critical adjunctive strategies to lower the risk of augmentation.

### 4.3 Potential intervention pathways targeting the gut microbiota

As the connections between RLS, the gut microbiota, gastrointestinal disorders, and iron metabolism gain attention, microbial intervention has begun to be discussed (Xiao et al., 2023). However, based on current evidence, its value likely lies in improving the intestinal environment to facilitate iron utilization rather than acting as a direct replacement for iron supplementation (Iddrisu et al., 2025). The gut microbiota influences luminal iron bioavailability, mucosal barrier status, and inflammatory levels; therefore, for patients with dysbiosis or gastrointestinal dysfunction, adjusting the microecology could theoretically act on both iron absorption and systemic distribution processes (Liu et al., 2024). For example, increased production of short-chain fatty acids helps maintain the local metabolic environment and intestinal barrier integrity, while the reduction of gut-derived inflammation may lower IL-6 and hepcidin-mediated iron restriction (Song et al., 2022). Existing studies have shown that certain probiotics, prebiotics, or dietary interventions can improve iron absorption or status, but due to the high variability in study subjects, intervention methods, and outcome measures, a definitive protocol applicable to RLS has yet to be established (Husmann et al., 2022).

From the perspective of RLS itself, the most noteworthy aspect of microecological intervention may be its impact on treatment response. A subgroup of patients concurrently suffers from SIBO, IBS, or other gastrointestinal abnormalities, which may impair oral iron absorption and maintain a state of low-grade inflammation (Liu et al., 2022). Research has identified associations between RLS and SIBO/IBS, and recent microbiota studies have observed distinct microbial compositions in RLS patients compared to healthy individuals and those with insomnia (Wang et al., 2026). Recent microbiota studies have observed distinct microbial compositions in RLS patients, with significant decreases in *Lachnoclostridium* and *Flavonifractor* genera compared with both healthy controls and insomnia patients (Montini et al., 2026). Reduced SCFA-producing taxa in RLS have been speculated to link gut dysbiosis to systemic inflammation and iron dysregulation (Montini et al., 2026). Although these cross-sectional findings cannot prove that intestinal abnormalities are the cause of RLS iron metabolism disorders, they at least indicate that gastrointestinal status is a background factor influencing iron metabolism and treatment response in some patients.. This issue is particularly worthy of further research in patients with preserved ferritin levels who fail oral iron therapy or present with prominent gastrointestinal symptoms.

Currently, there is insufficient evidence to support the routine use of probiotics, prebiotics, antibiotics, or fecal microbiota transplantation for RLS (Ciurea et al., 2026). Existing research remains largely at the stage of disease association and mechanistic speculation; studies that simultaneously track microbial shifts, hepcidin levels, iron metabolic indices, and clinical symptoms are still lacking (Tesoi et al., 2026). Therefore, the focus of future research should not merely be comparing whether a specific microbial intervention can alleviate RLS symptoms, but rather clarifying whether it first improves the gut environment or inflammatory state, thereby downstream influencing iron absorption, mobilization, and central supply. Only once these intermediate steps are validated can we determine whether the gut microbiota is an active participant in RLS iron metabolism abnormalities or merely a concurrent finding.

## 5 Discussion and future perspectives

The mechanistic framework proposed in this review highlights the multidimensional nature of iron dysmetabolism in RLS, encompassing intestinal absorption, systemic distribution, BBB transport, and central cellular utilization. However, several critical limitations in the current evidence base must be acknowledged. The proposed connections between gut dysbiosis, systemic inflammation, and central iron insufficiency should be interpreted within a hierarchical evidence framework: the hepcidin-ferroportin axis and its regulation by IL-6 are well-established, whereas the direct causality linking specific microbial taxa to BBB iron transport dysfunction in RLS remains speculative and requires prospective validation. First, much of the evidence linking gut microbiota alterations to RLS pathophysiology remains correlational. While distinct gut microbiome signatures have been observed in RLS patients compared to healthy controls and those with insomnia (Montini et al., 2026), these cross-sectional findings cannot establish causality. It remains unclear whether gut dysbiosis is a primary driver of iron metabolic disturbances, a consequence of systemic iron dysregulation, or an epiphenomenon confounded by comorbidities such as obesity, dietary habits, or medication use. Second, imaging studies on brain iron content in RLS have yielded discordant results. While some QSM and T2 relaxometry studies have identified decreased iron content in the thalamus, dentate nucleus, or substantia nigra (Li et al., 2016), others have failed to detect significant group-level differences (Kim et al., 2023). These discrepancies may arise from differences in MRI techniques, patient phenotypes (early-onset versus late-onset), medication status, or the heterogeneous nature of RLS itself. The lack of standardized imaging protocols and reference values across studies further complicates data interpretation. Third, the precise causal sequence linking peripheral iron status, BBB iron transport, and neuronal iron utilization remains unresolved. Although CSF from RLS patients has been shown to impair BMEC iron uptake *in vitro* (Palsa et al., 2026), whether this defect is primary or secondary to systemic inflammatory or metabolic signals is unknown. Similarly, while elevated hepcidin levels have been observed in RLS patients (Dauvilliers et al., 2018), the upstream triggers—whether gut-derived inflammation, adiposity-related signals, or other sources—have not been definitively identified.

These limitations point to clear research priorities. Longitudinal cohort studies are urgently needed to track the temporal relationship between gut microbiome composition, systemic iron parameters, hepcidin dynamics, and RLS symptom progression over time. Such studies should incorporate multi-omics approaches integrating metagenomics (to profile microbial functional capacity), metabolomics (to quantify SCFAs, tryptophan derivatives, and other bioactive microbial metabolites), and iron metabolic indices (serum ferritin, hepcidin, TSAT, and CSF biomarkers where feasible). This integrative strategy could identify specific microbial taxa or metabolic pathways that consistently associate with iron dysregulation and symptom severity, potentially revealing actionable targets for intervention. Furthermore, stratified clinical trials are warranted to evaluate iron supplementation efficacy according to pathophysiological phenotypes—for instance, distinguishing patients with absolute iron deficiency from those with hepcidin-mediated functional iron deficiency or those with predominant BBB transport defects. Such phenotyping would enable more precise allocation of oral versus intravenous iron therapy and could identify patient subgroups most likely to benefit from adjunctive microbiome-targeted interventions. Finally, mechanistic studies using human induced pluripotent stem cell (iPSC)-derived BMEC models or organoid systems could help elucidate the molecular pathways by which inflammatory signals and microbial metabolites influence TfR1 expression and iron transport across the BBB, providing a platform for drug discovery targeting the hepcidin-ferroportin axis or BBB iron transporters. Specifically, whether microbiota-derived metabolites such as SCFAs can modulate TfR1-mediated iron uptake at the BBB in RLS warrants direct experimental validation, building on existing evidence for SCFA-mediated BBB protection in other neurological conditions (Fock and Parnova, 2023).

## 6 Conclusion

In summary, iron metabolism dysregulation in RLS is a multidimensional disorder spanning intestinal absorption, systemic distribution, BBB transport, and central subcellular utilization. Peripheral indices such as serum ferritin often fail to reflect true central iron status, as dissociation between peripheral and central iron metabolism stems from hepcidin-ferroportin axis-mediated distribution constraints and functional impairments of transport receptors within BBB endothelial cells. Disruptions at these checkpoints deprive neurons of bioavailable iron and disrupt the homeostatic balance of dopamine, adenosine, and glutamate, triggering the sensorimotor symptoms and sleep disturbances characteristic of RLS. Additionally, the gut microbiota serves as an upstream environmental modifier of host iron metabolism; intestinal dysbiosis and gut-derived inflammation can activate the IL-6/hepcidin pathway, exacerbating functional iron deficiency. Given the profound heterogeneity of iron metabolism in RLS, future clinical interventions must emphasize targeted and individualized approaches that incorporate assessments of iron mobilization efficiency and central delivery capacity, guiding rational selection of oral or intravenous iron formulations to circumvent absorption and transport barriers.
